# Uterine Polypi

**Published:** 1883-09

**Authors:** William Goodell

**Affiliations:** Professor of Gynecology in the University of Pennsylvania


BUFFALO

### Medical and Surgical Journal.

VOL. XXIII. SEPTEMBER, 1883. No. 2.

### Original Communications.

### UTERINE POLYPI.

A CLINICAL LECTURE DELIVERED AT THE HOSPITAL OF THE UNIVERSITY OF PENNSYLVANIA, JANUARY 17, 1883.

BY WILLIAM GOODELL, M. D.,
Professor of Gynecology in the University of Pennsylvania.

REPORTED BY WILLIAM H. MORRISON, M. D.

GENTLEMEN—I have often stated to you as a broad rule, that when a married woman, over the age of forty, who has borne children, comes to you giving a history of hemorrhage, the first thing that you should think of is carcinoma, and in nine out of ten cases that is what will be found. If, however, the woman is unmarried, or if married, has not borne children, a benign tumor of some kind will in all probability be found There may be occasional exceptions to this rule. Sometimes the bleeding will be due to a sarcoma, but this is rare, and may virtually be left out of consideration.

Last Saturday an unmarried woman came to my office with a history of hemorrhage, violent ovarian and uterine colic and spasm of the bladder. I said to myself, "this must be a tumor of some kind and not carcinoma." I placed her on the examining table and was about to make an examination, when it suddenly flashed across my mind that in two hours I had to perform an oöphorectomy, and there might be here a polypus which had broken down producing a fetid discharge which would infect my fingers, so I decided not to examine, and, after explaining the reason to the patient, I asked her to return on Monday. She came at the time appointed; I found a polypus, and told her to come to this clinic.

It might be well to here explain the difference between ovariotomy and oöpherectomy. They both mean removal of the ovaries; in ovariotomy the ovaries are removed because they are diseased; in oöpherectomy they are removed, not for disease of the ovaries, but for some extrinsic purpose, usually in order to bring on the change of life.

Here she is, a woman of about forty years of age, with an history that the monthlies became menorrhagic. When I pass my finger into the vagina, I feel in the os uteri a tumor, around which I can not pass my finger.

Polypi, probably all arise as submucous fibroid tumors, which start in the fibroid layer directly beneath the mucous membrane and grow in towards the uterine cavity, that being the direction of least resistance. Having reached the cavity, the peristaltic action of the womb in its endeavors to extrude the foreign body causes the formation of a pedicle. Polypi are therefore nothing more than pedunculated fibroid tumors of the womb. They may be found in three different positions: They may be (1) wholly in the vagina; (2) they may protrude from the neck of the womb, being partly within the cavity and partly in the vagina like the clapper of a bell, or (3) they may be entirely within the cavity of the womb. From the first situation they are easily removed except when very large. They may at times be almost as large as a child's head. Two years ago, I removed one which was so large that I had to make lateral incisions in the perineum, and even then there was a tear. After the removal she had all the evidences of having given birth to a child. When she left, I told her that if she had any trouble, she should write to me for a certificate of virginity, for previous to the operation I found a complete hymen. After she went home, there were such whisperings among the old women of the village, that I had to write out the certificate.

When the polypus is in the vagina, our plan is to twist it off, or if it is large, to remove it with the wire ecraseur, or even piece-meal, in the best way we can. If it is dangling from the mouth of the womb, we do the same thing. When, however, the polypus is in the cavity of the womb the direction given in the old English recipe for making hare soup is applicable, "first catch your hare." So here, one must first catch the polypus before one can remove it. The first thing is to determine whether or not there is a polypus inside of the womb; we have to infer it from the length of the womb; the sound will sometimes tell us at once. In order to be certain, you dilate the cervix by sponge tents and pass in the finger; if you discover a polypus, you must redilate the canal, if you have not already done so sufficiently, and then catch hold of it with the polypus forceps. This is a most valuable instrument; if I could not get its counterpart, I would not part with it for its weight in gold. In removing a stone from the bladder you know that the instrument is passed and the stone caught at haphazard. In polypus of the uterus, the same thing can often be done. I have frequently twisted off a polypus in the cavity of the womb when I did not know positively that one was present. The forceps have only to be opened to the width of the polypus plus the thickness of the blades, which is very slight. If the volsella forceps are used, the blades have to be separated fully half an inch more than the thickness of the tumor, in order that its fangs may secure it. This instrument, which is the one frequently advised, has other disadvantages; the womb may be caught instead of the tumor; and further, if after you have caught the polypus you find that it is tearing, it is a difficult matter to release it without injuring the womb; the fenestrated polypus forceps is therefore the instrument to use.

I have told you the three positions in which polypi are found, but there is a word of caution to be given. A polypus may be exactly mimicked by an inverted womb. Such wombs have been frequently removed, and that by the best surgeons, under the impression that they were polypi, for it is often difficult to determine whether a tumor projecting from the cervix of the womb is a polypus or an inverted womb.

How are you to decide? What are our landmarks? Inversion of the womb is rare; polypus is not rare. An inverted womb is small; a polypus is often large. An inverted womb may, however, be large because it has in its wall a fibroid tumor. An inverted womb is sensitive; a polypus is not. If, before the patient is etherized, you pinch or squeeze an inverted womb, pain will be complained of, but if the polypus is pinched, or a pin stuck into it, the patient will not flinch. An error may come in here that is, a fibroid tumor covered with mucous membrane might be extruded. That membrane is supplied with nerves, and any injury to it would cause pain. In such a case, the error would be on the safe side, for if the tumor were sensitive, you would perform no operation until you were thoroughly satisfied that the body was not the womb. Inversion of the womb is exceedingly rare in virgins and it is not at all common in married women. I have never seen an instance in a virgin. It usually occurs as the result of labor and is recognized at the time. It is then either replaced or the woman dies. The sound will aid in the diagnosis. There will not be a uterine cavity in inversion but if the womb measures two and a half inches, you may be sure that there is no complete inversion. Here comes a difficulty, and here it is where the best surgeons, and the most excellent diagnosticians, have made the mistake of removing a portion of the womb together with the polypus. The womb in trying to extrude the polypus, may become cupped, like the bottom of a wine bottle. The traction made by the surgeon will often do the same thing. He applies the wire of the ecraseur to what he supposes to be the pedicle, when in fact he has noosed the cupped portion of the womb which is removed with the tumor. It is not always easy to determine whether this cupping has taken place or not. You would be astonished to learn how many good sugeons have removed a portion of the womb with a polypus; many cases have not been reported, for if the result be fatal the case is less likely to be reported. On the other hand, I believe that in many instances a part of the womb has been removed, without the knowledge of the physician, who wondered why his patient died. In the present case, I have not been able to decide whether the tumor is a polypus or an inverted womb, for when I attempted to pass the sound, I could not discover a uterine cavity. I said to this patient, "now I want you to tell me one thing, have you ever borne a child?" This made her quite indignant and from her expression I feel certain that she is a virgin. To-day she is under ether, which makes matters easier.

I shall try to draw the womb down. This will facilitate diagnosis. The pedicle is either fastened to the sides of the os by inflammatory lymph, or else some portion of the womb is inverted. If the woman is a virgin, as I believe she is, the latter is hardly possible, but it does sometimes occur. I tried to pass the sound in my office but did not succeed, but I have now broken an adhesion and the sound enters three inches into the cavity of the womb. I also feel the fundus above the pubes. My conclusion, therefore, is that a fibroid tumor has gradually been forced from the wall of the uterus into its cavity and thence into the vagina, without becoming pedunculated. From the measurement given by the sound I know that the greater part of this tumor is outside of the womb, but it is impossible to say where the tumor ends and the womb begins. I shall therefore try to enucleate the tumor. It is covered by mucous membrane. I slit this membrane and strip it off. In this way I shall take out the tumor without injury to the womb. It comes off, you see, with some degree of ease but not as readily as the rind from an orange. This is precisely the way in which fibroid tumors within the cavity of the womb are enucleated. Where no pedicle exists, this is the safest way of operating. In enucleating a tumor inside of the uterus I use an Adams's subcutaneous saw to make an opening in the most projecting part of the tumor. I prefer the saw because it is easier to manage in such cramped quarters than the scissors or a probe-pointed knife. Further, the bleeding is less and points can be reached by it that are inaccessible to the other instruments. I make an opening into the capsule as large as possible, sometimes not more than an inch, sometimes two or three inches in length. I then pass my finger and peel off the mucous membrane as far as I can. If possible, the tumor should be removed at one sitting; but if this cannot be done, ergot should be given to induce contractions of the uterus. In a few days, the little wound will be forced open by the tumor and you can then proceed with its enucleation.

I have now removed the tumor. It was not pedunculated, but, as I said, is a fibroid in the process of self-enucleation. I have simply completed what nature had begun. I shall now examine the womb to see whether it has been at all inverted. It is not, and as there is no bleeding I shall do nothing more. Should bleeding occur in such an operation, vinegar is the best haemostatic. I never use Monsel's solution unless driven to it by an alarming hemorrhage. The objections to it are that it produces plaster-like clots; these remain inside of the womb, break down with a bad odor and are liable to cause septicæmia. It is not a safe haemostatic in regions where the tissues are erectile, as about the womb. In such situations, the veins and lymphatics staying open are more likely to absorb septic material than in any other portion of the body where these vessels contract after being cut. Another objection to the use of Monsel's solution is that it so corrugates and contracts the vagina as to interfere with the needful manipulation. Do not, therefore, use Monsel's solution in operations on the womb. I have often used it in cancer of the cervix, and have never had any bad results, for in this condition there are friable granulations which need corrugation. As this patient will remain in the hospital where she can be watched by the physicians and nurses, I shall not plug up the wound. If I performed this operation in the country where I could not see the patient for several hours, I should cram a sponge into the cavity which formed the nidus of the tumor. I do not like to plug the womb because the blood collects behind the plug and clots. In a few hours it begins to putrefy and give off an odor; in 12 hours the smell is more decided, and in 24 hours it is exceedingly offensive.

There is another variety of polypus of which I neglected to speak. It is a small affair which might seem to you insignificant, and yet has destroyed life. I refer to an enlarged nabothian gland which has become pedunculated. It will be found either partly within the canal and partly without, or just within the os uteri. The finger will usually detect it, but being very small it sometimes retreats within the canal before the examining finger, and the speculum may be necessary for its discovery. Women have actually bled to death from the presence of a small mucous polypus, or a pedunculated nabothian gland, not larger than a pea.

The after treatment of our patient will be simply to keep the patient sweet and clean by vaginal injections of potassium permanganate. Her bowels will also be kept bound for a few days, and she will stay in bed until all vaginal discharges from the sore have ceased.

